# Medial Discoid Meniscus: A Rare Case Report

**DOI:** 10.7759/cureus.39971

**Published:** 2023-06-05

**Authors:** Ziyad S Al Saedi, Bandar K Alzubaidi, Hashem A Mirza, Mohammed K Alhothali, Majed M Alhijjy, Amr A Mirza

**Affiliations:** 1 Faculty of Medicine, Umm Al-Qura University, Makkah, SAU; 2 Orthopedic Surgery, King Fahad General Hospital, Jeddah, SAU

**Keywords:** pain, knee, arthroscopy, saucerization, orthopedic, medial discoid meniscus

## Abstract

The meniscus is a glossy white structure found in the knee between the femoral condyle and tibial plateau in the medial and lateral aspects of both knees. The main purposes of the meniscus are to enhance joint congruity and stability, transmit load, and absorb stress. A rare type of anomaly of the meniscus shape is called discoid meniscus, which presents as an atypical shape also known as disk cartilage. This report presents a 13-year-old male with a history of left knee pain after a fall. The pain was stabby in nature with a decrease in range of motion in the left knee and positive McMurray and Apley’s tests on examination. The patient was treated by arthroscopic saucerization, and the procedure was successful. The patient had a good postoperative outcome after two months of follow-up.

## Introduction

The meniscus is a glossy white structure found in the knee between the femoral condyle and tibial plateau in the medial and lateral aspects of both knees. The main purposes of the meniscus are to enhance joint congruity and stability, transmit load, and absorb stress. Some studies have also hypothesized that the menisci play a role in proprioception and joint lubrication. Although the lateral menisci are more variable in size, shape, thickness, and mobility than the medial menisci, both menisci are generally wedge-shaped and semi-lunar [1,2].

A rare type of anomaly of the meniscus shape is called discoid meniscus, which presents as an atypical shape also known as disk cartilage. It is a special type of meniscus that is thicker and covers a larger area of the tibial plateau than a standard meniscus. The characteristics and epidemiology of discoid meniscus are still poorly understood, but it is known that the discoid lateral meniscus is more common than discoid medial meniscus. The reported incidence rates for discoid medial meniscus range from 0.12% to 0.3%, while those for discoid lateral meniscus range from 1.2% to 5.2%. The discoid medial meniscus is therefore considered very rare, although the real incidence of discoid meniscus is difficult to measure because many patients are asymptomatic [3-5].

We report here on a rare case of a symptomatic ipsilateral discoid medial meniscus in the left knee of a 13-year-old child after a falling injury.

## Case presentation

We present the case of a 13-year-old male patient presented to the orthopedic outpatient clinic complaining of left knee pain with a limited range of motion for one year.

History of present illness

One year ago, the patient fell on his left knee, after which he began to experience pain in the left knee. The patient described the pain as stabbing in nature, with no radiation; the pain was exacerbated by squatting, flexing the knee, and walking for a long distance. The pain was relieved by analgesia and rest. The patient mentioned different scales of pain, starting with 6/10 to unbearable pain if not rested; there was no fever and no morning stiffness. The patient has an unremarkable past surgical and medical history and an unremarkable family and genetic history.

Physical examination

**Figure 1:**
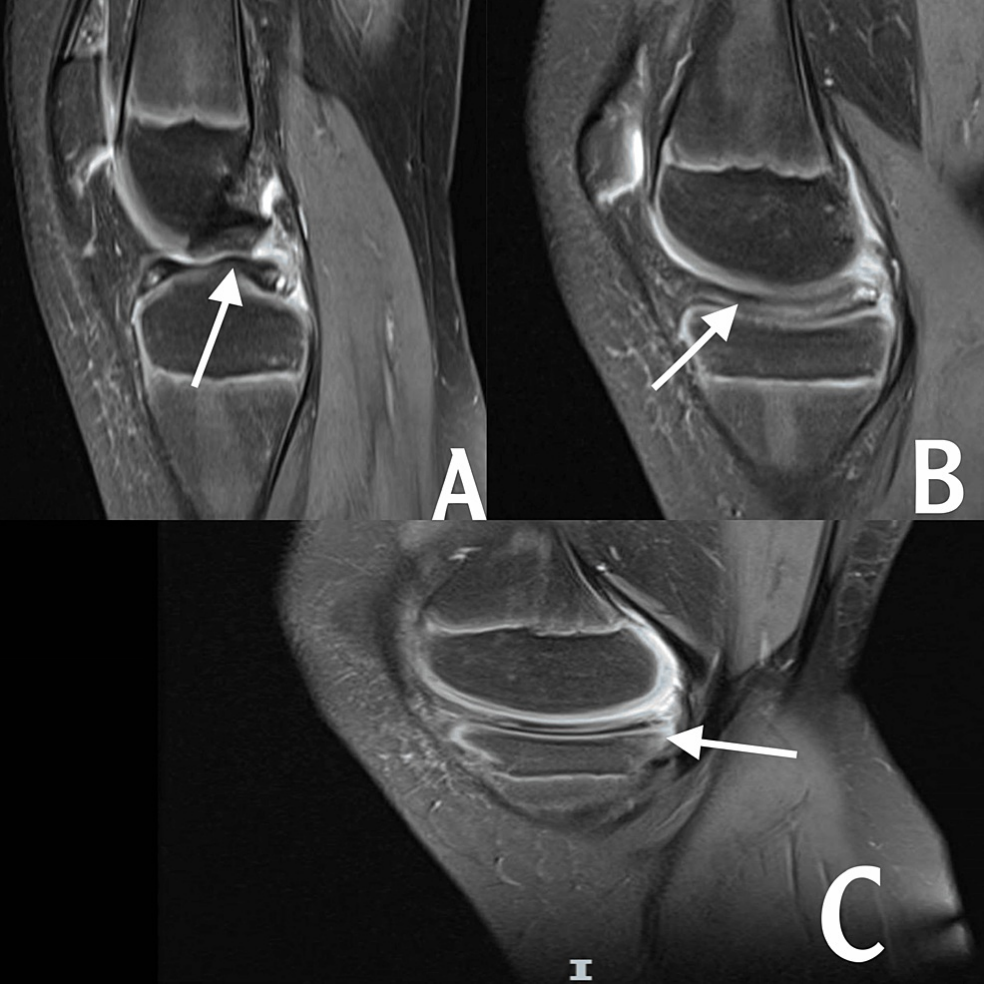
Left knee MRI sagittal view

**Figure 2:**
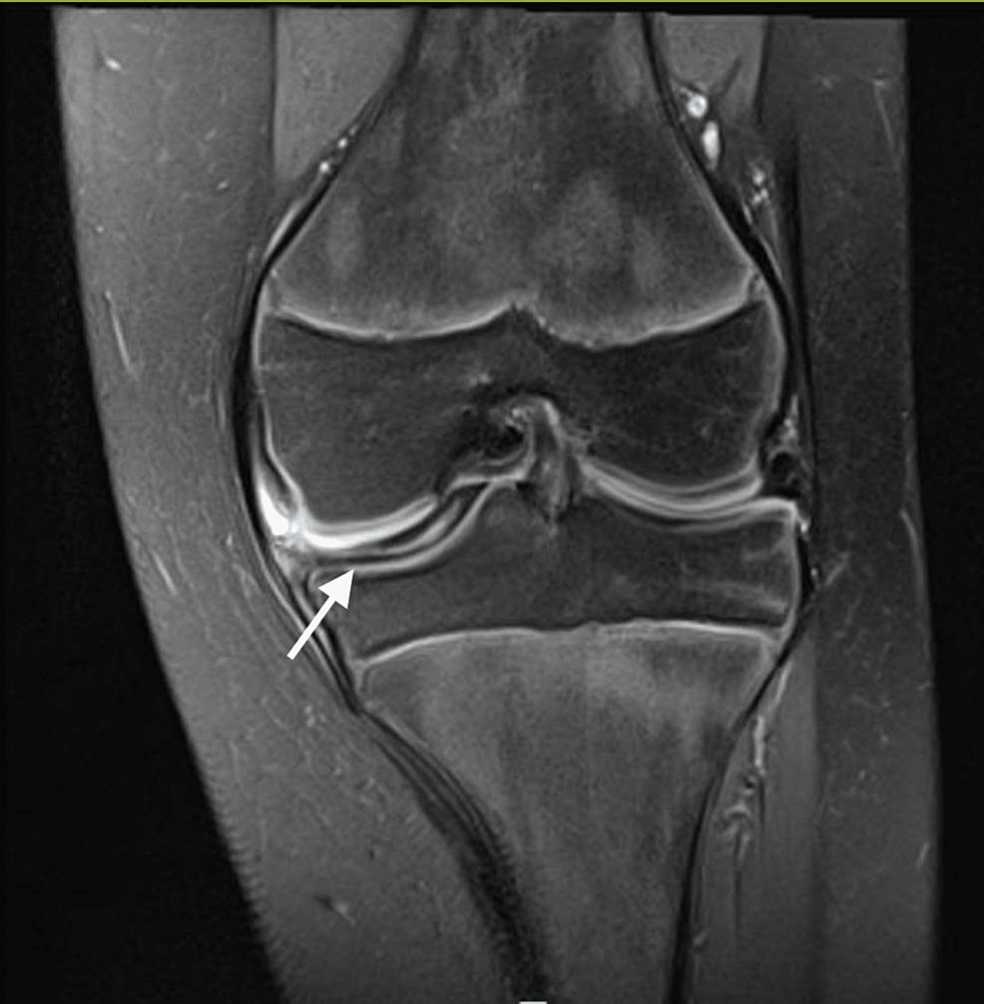
Left MRI coronal view

Upon physical examination, the patient’s muscles were normal and there was no knee swelling on either side of the knee. The temperature and skin color were normal bilaterally. The floating patella test was negative. The left knee showed mild limping, no redness or scars, and no valgus or varus deformity. On palpation, there was tenderness on the posteromedial aspect of the left knee. McMurray and Apley’s tests were positive in the left knee, with negative effusion, negative anterior and posterior drawer test, and negative Lachman test. The Lysholm score was 65, while the range of motion of the left knee was (0-120) upon extension, compared to the right knee (0-140), without any ligament instability. The sagittal MRI view of the left knee indicated a discoid medial meniscus with meniscus tear (Figure 1), and the coronal MRI view of the left knee showed the discoid meniscus reached to intercondylar notch and also an inter-meniscus tear (Figure 2).

Management

**Figure 3:**
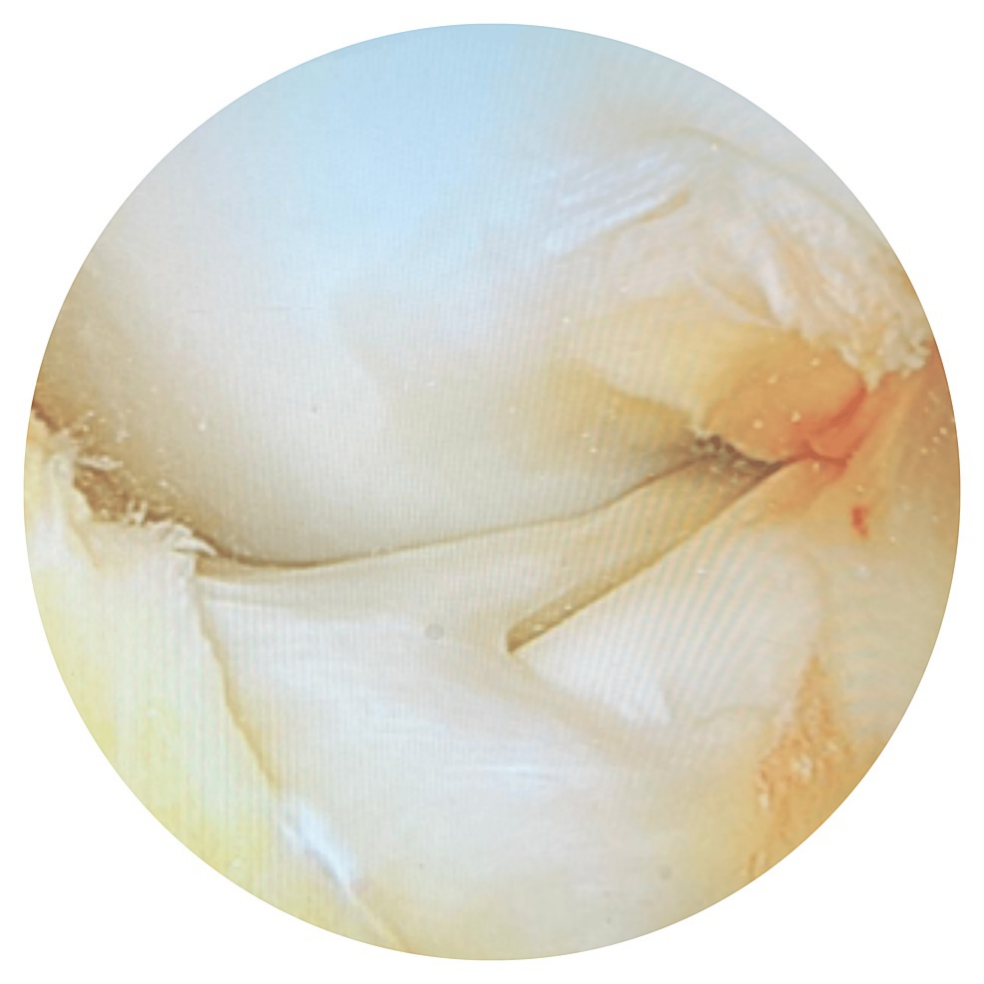
Medial discoid meniscus pre-saucerization; it reached to the intercondylar notch

**Figure 4:**
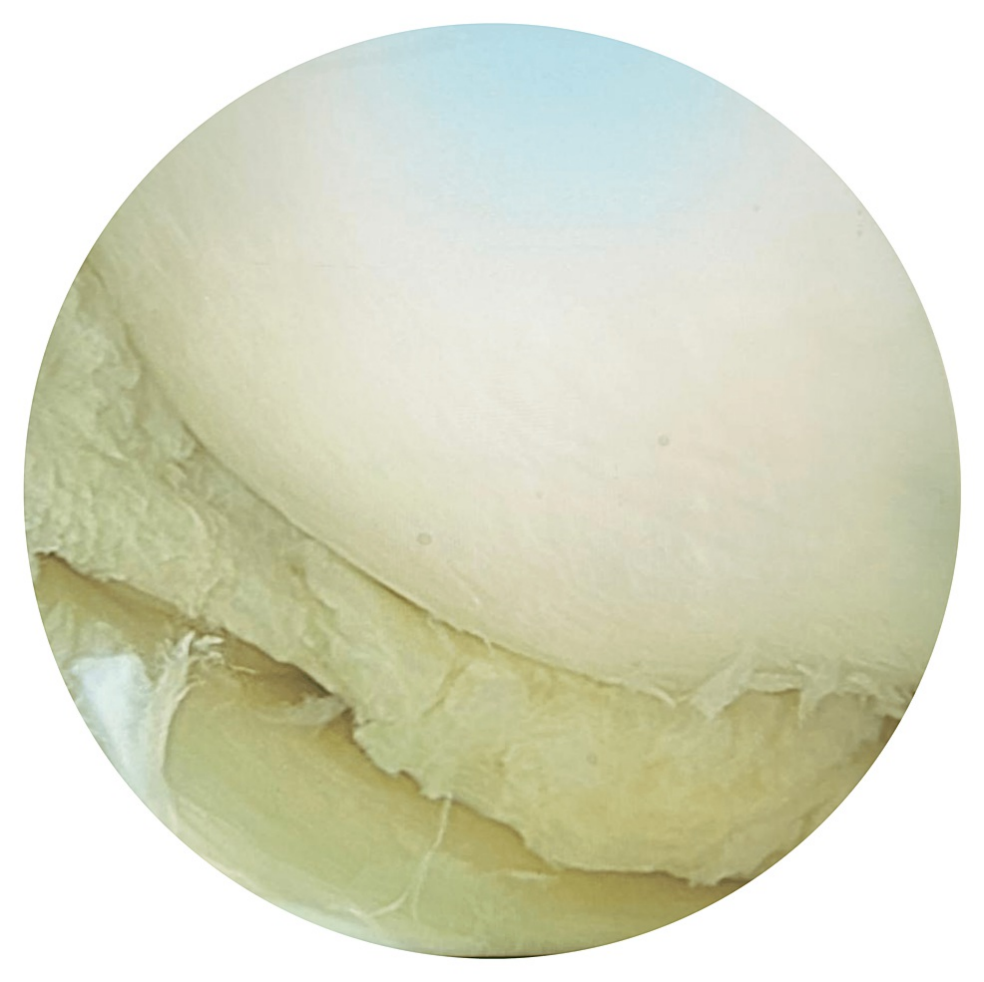
Post saucerization

After confirming the diagnosis of symptomatic discoid medial meniscus, the patient went for an arthroscopy of the left knee under general anesthesia. The arthroscopy showed a discoid medial meniscus, which reached the intercondylar notch (Figure 3). A saucerization of the discoid medial meniscus was performed (Figure 4), and physiotherapy sessions started after one week.

At the two-month follow-up, the patient’s pain and range of motion restriction had disappeared, and McMurray and Apley’s tests were negative, with complaint of slight pain after exercise and daily activity and a slight problem in squatting; the Lysholm score was 94.

## Discussion

A discoid medial meniscus is currently considered a very rare congenital abnormality. At present fewer than 80 cases have been documented in the literature [6]. This incidence may not be an accurate reflection, because the condition may be asymptomatic, and diagnosis may happen accidentally by MRI, with patients being advised to leave the condition untreated if there are no severe symptoms [3,6]. However, in the literature, some cases are reported as having different symptoms, such as locking and effusion, medial knee pain, a limited range of motion in the affected knee and clicking, but in our case, the patient complained of pain and a limited range of motion in the left knee after exertion [3,4,7].

According to the literature, the management of a symptomatic discoid medial meniscus consists of arthroscopic saucerization, and some cases are treated by partial meniscectomy [3,4,7-9]. If a horizontal cleavage tear is present, doctors prefer to do a partial meniscectomy over saucerization, specifically when the peripheral vascular zone is affected. To date, the clinical outcome of various forms of management of discoid medial menisci is still poorly understood due to its rarity. According to the limited literature, the short-term and intermediate outcomes have generally been positive [10]. The saucerization of the discoid lateral meniscus is better understood than the discoid medial meniscus, because it has a higher incidence. A retrospective of 11 cases that underwent saucerization (n = 2) reported good outcomes, and the patients (n = 11) reported excellent outcomes at 4.5 years after surgery [11].

Another study found that 44 out of 48 patients reported good to excellent postoperative results at the 10-year follow-up [12]. To date, our patient has had a complaint of slight pain at a two-month follow-up after surgery and a slight problem in squatting, indicating a satisfactory outcome. However, since the discoid meniscus has been identified as one of the risk factors for articular cartilage lesions, a longer follow-up period is necessary [13,14].

## Conclusions

In conclusion, medial discoid meniscus congenital anomaly in knee meniscus comes with discoid shape rather than normal shape and it may cause some symptoms like knee pain and decrease range of motion, but some patients have no symptoms at all. Medial discoid meniscus can be treated in different ways, like suture and partial meniscectomy. Our patient complained of knee pain and decreased range of motion on the left knee and was treated by saucerization with good postoperative outcome.
